# Reimagining research ethics to include environmental sustainability: a principled approach, including a case study of data-driven health research

**DOI:** 10.1136/jme-2022-108489

**Published:** 2022-08-03

**Authors:** Gabrielle Samuel, Cristina Richie

**Affiliations:** 1 Department of Global Health and Social Medicine, King's College London, London, UK; 2 Philosophy and Ethics of Technology Department, Delft University of Technology, Delft, Netherlands

**Keywords:** research ethics

## Abstract

In this paper we argue the need to reimagine research ethics frameworks to include notions of environmental sustainability. While there have long been calls for health*care* ethics frameworks and decision-making to include aspects of sustainability, less attention has focused on how *research* ethics frameworks could address this. To do this, we first describe the traditional approach to research ethics, which often relies on individualised notions of risk. We argue that we need to broaden this notion of individual risk to consider issues associated with environmental sustainability. This is because research is associated with carbon emissions and other environmental impacts, both of which cause climate change health hazards. We introduce how bioethics frameworks have considered notions of environmental sustainability and draw on these to help develop a framework suitable for researchers. We provide a case study of data-driven health research to apply our framework.

## Introduction

Dominant research ethics paradigms often revolve around ethics principles that are concerned with the protection, rights, safety and welfare of *individual* research participants. These paradigms can be traced back to a number of historical ethics frameworks developed in response to atrocities in biomedical/clinical research in the 20th century,^1^ and include the 1968 Declaration of Helsinki^1^ and the subsequent 1979 Belmont Report.^2^ These frameworks aim to guide physicians and researchers in appropriate clinical research ethics conduct, with relevant ethical principles including the need for research to respect individual research participants in group or individual settings; the need to ensure that research design minimises individual risk while maximising potential societal benefit; and the need to ensure fair practices in the selection of individuals for participation in research studies.

While individualised risk has long been a focus of research ethics frameworks, strong criticism exists around it. In an interconnected world it is difficult to argue that the impacts of individual research treatment would not affect others, particularly in the closer communities of friend and family groups. Carol Gilligan’s work on care ethics^4^ and the notion of relational autonomy both point to the networks that impact ethical decision-making within healthcare. Furthermore, concerns have long been raised about the appropriateness of placing individual risk ahead of *communitarianism*, especially in research areas that are less concerned with individual health, such as global health research. Public health scholars have long pointed to the moral status of the community in research ethics considerations,^5–8^ whereby community harms are more than the sum of individual values and interests and relate to questions associated with whether communities will be beneficiaries of the research, or even whether they share the same goals as the researchers.^9–11^ Multiple authors have pointed to the abusive practices and problematic studies conducted with tribes, indigenous populations, and minoritised and marginalised communities worldwide over the past decades, which have failed to consider community harms associated with violating widespread trust or taking ownership of a community’s stories.^10^ For these reasons Emmanuel and Weijer^9^ emphasise the importance of an ethical principle of ‘respect for community’ alongside more individual principles related to risk and exploitation, such that scholars need to devote careful attention to understanding the sociopolitical impact of research on communities as a whole and not only to individuals,^7 12 13^ remembering that individuals are part of the whole community.

While concerns about community harm have expanded moral status considerations beyond those focused on individual risk alone, they are anthropocentric and have stopped short of considering environment-related harms associated with the research process. The environmental impact of the medical industry and health research can be measured by carbon emissions and resource use. The carbon emissions of global healthcare activities, including research, make up 4%–5% of the total world emissions.^14^ The *Lancet* reports that the Sustainable Clinical Trials Group calculated nearly 350 000 national and international trials registered on ClinicalTrials.gov ‘using the average…(to) give a carbon consumption of an estimated 27.5 million tonnes, which is just under a third of the total annual carbon emissions of Bangladesh, a country of 163 million people’.^15^ The impact of carbon emissions includes not only climate change, but also health hazards like pollution, significant environmental destruction, use of scarce resources, loss of biodiversity and diminished quality of life for humans.^16^ People affected by climate change require medical care, which is predicated on medical research.^17^ These treatments release more carbon, locking healthcare into a self-destructive cycle whereby medical research, care and treatments cause medical needs. Hence, healthcare research has a special interest in carbon reduction, not only as a matter of international priority, but also as a commitment to health. In this paper we draw on the concept of sustainability to provide an ethical basis for the inclusion of such environmental harms in health research.

## Environment and (bio)ethics

In 1927 Fritz Jahr described bioethics (German: *bio-ethik*) as ‘the assumption of moral obligations not only towards humans, but towards all forms of life’.^18^ Jahr drew on Rudolf Eisler’s *Bio-Psychik*, declaring: ‘Respect every living being on principle as an end in itself and treat it, if possible, as such!’ (p230). Almost half a century later in 1971, the term ‘bioethics’ appeared in English with a parallel scope when Van Rensselaer Potter used it to describe a life-ethic for an industrialised society in a precarious ecosystem. For Potter, bioethics was rooted in an intrinsically practical approach to ecologically sustainable life, inclusive of the earth and other organisms.^19 20^ Despite bioethics’ environmental origins, since Beauchamp and Childress’^21^ 1979 proposition of ‘biomedical ethics’, which focused on the patient–physician relationship through four principles of respect for autonomy, beneficence, non-maleficence and justice, ‘bioethics’ has become widespread conflated with ‘biomedical ethics’. This has erased the ecological origins of bioethics while simultaneously giving rise to the ‘new’ discipline of environmental bioethics.^22^

Nevertheless, an increasing number of scholars have advocated bioethics readopt a broader perspective that aims to explore the relationships between individuals and the natural environment.^23–29^ They reject that the land and ecosystems are just instrumentally valuable—good because of how humans can use them—but rather argue that our moral sentiments need to extend to the biotic community, to the soils, waters, plants and animals that make up our planet^30^ since nature is both inherently valuable—good in itself—and because humans are a part of, not separate from, nature.^30^ Most widely recognised ethical theories acknowledge interconnectedness (with people and communities), and it makes moral sense to include the biotic community within this moral framework.^31^ They call for a systems approach that considers individuals, populations and environmental factors in understanding (health) practices and policies (for instance, see Lee 25; also see Richie^32^).

Some effort has ensued in the research ethics community in this regard. The European Commission’s *Ethics for Researchers*—designed for researchers who are preparing an application for research funding from the European Union—includes respect for biodiversity, the environment and ecological balance as one of its 12 golden rules to ethical research conduct.^33^ Equally, the All European Academies *Code of Conduct for Research Integrity* points to the need not to ‘waste resources and [expose the] environment to unnecessary harm’ during research.^34^ The National Institutes for Health Research (NIHR) *Carbon Reduction Guidelines* ‘highlight areas where sensible research design can reduce waste without adversely impacting the validity and reliability of research’.[2] Similarly, the UK’s research funding body, UKRI (UK Research and Innovation), emphasises that ‘public funds should be deployed with due consideration to value for money and environmental impact across all activities’.^35^

At the same time, a recent review of international research ethics frameworks by RAND suggests that such environmental concerns are primarily applied in non-human-centric disciplines; within human participant research, harm is generally considered anthropocentrically in human terms only.^36^ If moral reflections are to consider the environment, key unanswered questions include how we should give respect to non-human worlds, especially since human endeavours will always inevitably lead to the destruction of at least some of the biotic community and ecosystems, and how this respect should or could be weighed next to humans (p235).^37^ [3] Despite this, moral obligations to the environment still exist, even if they are anthropocentric and instrumentalising for reasons of self-preservation. The planet and its ecosystems sustain us. Without these ecosystems, humans can neither survive nor flourish,^37^ and indeed the destruction of our ecosystem has led to a diminished quality of life for billions of people, including early death, increased morbidity and psychological suffering.^38^

In the following section we argue that in research ethics frameworks, moral decision-making should extend to the environment. Drawing on the concept of sustainability, we map out what such a research ethics framework would look like.

## A research ethics framework based on sustainability

As scholars in healthcare increasingly shift to a broader vision of bioethics and take into account factors associated with non-humans and ecosystems, sustainability has become an important concept.^27 31 32 39–47^ Following from the well-cited ‘Brundtland Report’, sustainability is viewed as a forward-looking concept for guiding a wide variety of choices that are grounded on the commitment to the well-being of both current and future populations.^48^ ^4^

In her work on green bioethics, Richie^26^ draws on environmental ethics to propose a green bioethics framework for evaluating the sustainability of medical developments, techniques and procedures. This framework includes four normative principles: distributive justice takes a broad view of the moral community and requires the allocation of basic medical resources before special interest access; resource conservation to provide healthcare needs before healthcare wants; simplicity to reduce dependence on medical interventions; and ethical economics to promote humanistic healthcare instead of financial profit.^26^ We draw on this and other frameworks of restraint and justice from environmental bioethics (eg, see Potter and Lisa^49^ in Jameton and Pierce^31^). We modify it to be more aligned with current research ethics frameworks (eg, see Weinbaum *et al* 36 and Emanuel *et al* 50), thus making it intelligible and persuasive for researchers. In the following sections we map our research ethics framework of five substantive ethics principles: social value, scientific quality, respect for persons, communities and environment, justice, and favourable risk to benefit ratio.

### Scientific quality

Proposed research must be conducted in a methodologically rigorous manner, using reliable and valid research design and methods.^51 52^ Special attention to possible sample bias or underpowered research is important. Execution of the study is also important to ensure results are valid and answer the research question. A lack of quality leads to wasted resources and time. All research has a carbon footprint even if the results of the study are not published, or unusable for reasons of lack of replicability or lack of reproducibility. Hence, the NIHR suggests a thorough literature review prior to developing a research proposal.[5]

### Social value

Research must be beneficial to the participants, community, society^50 51^ and environment. More than just refraining from harming the individual, community, society or environment, it should proactively lead to improvements in health, the environment or well-being, or act as a preliminary step towards this. Anything short of this could expose individuals to harms without there being a worthy pursuit (especially if clinical research), or more broadly divert resources from other valuable pursuits. Since all research requires resources, maximal benefits should be prioritised since the consequence of research is increased carbon emissions and risks of climate change health hazards.

### Respect for persons, communities and environment

Respect for persons extends further than respect for autonomy, and considers one’s moral attitude towards others and the actions towards others that result from and exemplify this attitude.^53^ Respect for communities allows a broadening of this concept to include a variety of cultural norms, including those which place less emphasis on individual autonomy and autonomous decision-making than is the norm in some cultures.^54^ Procedural principles to help with respecting persons and communities include, for example, the need for trustworthiness, transparency, privacy and ownership, accountability, autonomy, engagement, the need for consent, and the right to withdraw.^36 51 53^ Respect for the environment includes taking environmental destruction into consideration by considering the environmental impacts associated with the research endeavour, particularly when that destruction occurs in places which may not directly benefit from the outputs, for example, clinical trials in the developing world, or in places where natural resources are used, not replenished and not properly compensated for (eg, harvesting of medicinal plants in a rainforest, mining).

### Justice

This has historically referred to fair participant selection based on the scientific goals of the proposed research.^50 51^ This also refers to the fair treatment of individuals and communities beyond research-based activities to ensure that those individuals or communities who take part in research are those most likely to benefit. It also refers to environment-associated harms and benefits associated with the research endeavour. This adheres to Nancy Fraser’s^54^ work on justice, which proposes an ‘all subjected principle’, such that ‘all those who are subject to a given governance structure have moral standing as subjects of justice in relation to it’ and that ‘for any such governance structure, the all subjected principle matches the scope of moral concern to that of subjection’. Brock’s work is useful here too. She sees a role for both state-bound *and* global justice when considering duties in healthcare.^55^ She explains that we should give special attention to those within our own state, but we have a moral obligation to make low or reasonable modifications to our own governance structures because of the negative duty to refrain from harming others. Following this premise, if low or reasonable modifications to our own governance structures would decrease harm caused to others, we have a moral responsibility to make these modifications. This is particularly pertinent for people living in affluent countries and their obligations for those who live in extreme poverty in developing countries, and particularly links to the risk to benefit ratio principle that requires finding the optimum research methodology that allows these risks to be minimised.[6]

### Favourable risk to benefit ratio

This is a key aspect of research ethics frameworks that is also related to principles of proportionality, beneficence and non-maleficence. Historically, a favourable risk to benefit ratio involves weighing the individual risk versus individual and/or collective benefit from the research in a utilitarian way (and more recently assessing community risk/benefit). To be truly utilitarian, and to consider all links within a consequentialist pathway, risk to benefit ratios must include environment-related risks.^31^ Jameton and Pierce^31^ argue that when these harms are put into the research ethics risk/benefit balance, ‘everyday decisions unquestioned by ethicists and regarded as rational and even praiseworthy may be seen as questionable and possibly maleficent’ (p119).^31^

Our proposed principles have direct relevance for health research. In the next section, we present a case study and then apply the principle to demonstrate the feasibility and agility.

## Case study: data-driven health research

Health research is becoming increasingly data-intensive. Through the capture and analysis of vast swaths of clinical, imaging and genomic data, other biomarkers, as well as data from wearable devices, social media and environmental exposures, researchers aim to improve detection, diagnosis and treatment of patients and the public. While data-driven health research and any technologies that emerge are viewed as a panacea towards better health and healthcare, they have adverse environmental impacts. This is because they rely on digital infrastructures that are not ‘virtual’ as implied by the metaphors describing them, but have materiality—they involve mining, manufacturing, transport, use and waste, all of which have carbon emissions, and all of which produce toxic and hazardous chemicals as well as other environmental and public health impacts. For health research approaches that rely on artificial intelligence (AI), such as diagnostic tests and healthcare disease prediction, we know that the largest AI models are doubling in necessary compute every 3–4 months, thereby severely outpacing the increasing efficiency of hardware.[7] Mining and e-waste also have associated environmental, health and well-being harms.^56 58^ For example, unregulated resource recovery from e-waste landfills has led to the generation of hazardous by-products shown to be present in those living around informal e-waste sites, at levels vastly exceeding recommended safety levels (see Gabrys^59^ and Ngo *et al* 60).

Over the past decades, the digital sector has worked hard to drive efficiency gains.[8] However, the most recent estimate of the sector’s contribution to global carbon emissions has been calculated between 2.1% and 3.9% global emissions.^61^ While health research only comprises a small proportion of all digital technology, health is the fastest growing sector in the datasphere^62^ and will become an increasingly important contributor, with proteomics, metabolomics and genomics all data-intensive solutions. Communication and media scholar Mel Hogan emphasised that by 2025, between 100 million and 2 billion human genomes will have been sequenced globally, using some 40 exabytes of data.^63^ The UK 100,000 Genomes Project, which has sequenced 100 000 genomes, is 21 petabytes,^64^ and by 2025 the UK Biobank database—a leading biobank internationally—is expected to grow to 15 petabytes, an amount of data equivalent to that created annually by the Large Hadron Collider.[9]

Moreover, as other sectors decrease their environmental impacts, the digital sector, including the digital aspect of health research, will increase consumption as it acts as an enabling technology. Backfire is also a concern, whereby the move towards increased digital efficiency, without constraints, results in more, not less, consumption. For example, app-based ridesharing increases use of vehicles instead of carbon neutral forms of transportation like walking and biking, thus ‘cancelling out 68% to 77% of CO_2_ emission reductions and 52% to 73% of aggregated social benefits (including congestion, air quality, carbon dioxide emissions, noise) expected from ridesharing’.^66^ While increasing the efficiency of digital technologies has historically been drawn upon as a solution to increased consumption, these efficiency gains are slowing.

The move to renewables is also only a partial solution because of its large dependency on mining, as well as its poor recycling prospects. Finally, while health research promises to lead to better health, there is often a lack of clarity about *whose* health and whether those who will benefit are those who are already experiencing greater access to healthcare. For those not receiving these benefits, health research may amount to only health *risks* in the form of environmental impacts.^67^

In the following sections we map out how researchers, ethicists and healthcare professionals can think about these issues through our principle-based research ethics framework.

### Scientific quality

Data should not be collected and analysed without ensuring that the research outputs will be of sufficient quality (considering issues of bias, etc). The storage and processing of data are not harm-free and should only be collected and/or analysed if there is an appropriate reason for doing so, such as translatability to significant medical progress, deep gains in knowledge, and the potential for widespread and just dissemination of any developments.

### Social value

Research should cobenefit humans, communities, society and environment. Social value could mean prioritising more low-tech research rather than energy-hungry data analyses, especially when low-tech research is likely to produce positive health benefits that are equal or greater than high-tech. For example, addressing social, economic, commercial and political determinants of health is likely less impactful on the environment. This is because it is often based on preventive medicine and low-tech interventions, rather than high-tech, reactive solutions that may only lead to benefit for the few who have access to medical infrastructures and sophisticated medical care.

### Respect for persons, communities and the environment

For data-driven health research, respect for persons and communities entails respecting all of those affected by the research. It involves community and individual engagement, the availability of readable and digestible information, transparency on how the data are regulated and the protections in place for individuals and communities whose data may be used, and accountability pathways.^53^ This can be collected and published online in an easily searchable database. Moreover, how this is used should be part of open-access articles and reports for the benefit of those in the broader scientific community.

Respect for the environment includes awareness of the environmental impact of the research and taking steps to reduce this. At one level, this could involve, for example, optimising algorithms to ensure they have as minimal impact on resource use and carbon emissions or choosing data centres with considerations of sustainability in mind (eg, if the energy they use to power them is ‘dirty’ or ‘clean’, non-renewable or renewable). A range of calculators can help researchers assess the environmental impact of their data-driven practices, and there are various guidelines and frameworks to assist.^68^ At a higher level, as researchers use more data, consumption and environmental impact will increase and this must be considered. Respecting the environment means minimising our data use as much as feasibly possible.

### Justice

For data-driven health research, this refers to, for example, the fair collection, storage, use, linkage and sharing of data,^53^ as well as attention to equity and benefit sharing of research outcomes. Consideration must also be given to environment-related harms. This includes those involved in mining minerals used in digital technologies, manufacturing them and recycling/disposing of them. This also includes aspects of social justice, for example, questioning the inequalities associated with the use of turks to analyse data. Justice must also consider how research results will be used in terms of the long-term implications and carbon expenditures.

### Favourable risk to benefit ratio

Risk to benefit ratios need to include weighing up individual, community and environmental risk against benefit. As historically noted, this decision will include some measure of subjectivity, but overall should focus on minimising harm as much as possible. This can be achieved by, for example, buying repurposed machines where possible, using data centres that are powered by renewables and having appropriate recycling infrastructures for digital technologies. However, reliance on ‘recycling’ still requires resources. Hence, the familiar environmental manta ‘reduce, reuse, recycle’ is relevant: recycling should be the last resort on the path to sustainability, not the default.

## Conclusion

As the levels of atmospheric carbon are already over safe levels of 350 parts per million,^69^ research must be done parsimoniously in ways that neither suppress scientific invention and creative nor threaten the health of people and the planet. We have mapped out a research ethics framework that allows us to do this.

## Footnotes

Such as World War II, the Tuskegee Syphilis Study and the Henrietta Lacks case. The Tuskegee Syphilis Study was a longitudinal study conducted by the US Public Health Service in Tuskegee, Alabama, in which approximately 600 African Americans participated between 1932 and 1972. In 1972 it was revealed that the participants had received a dishonest explanation for their involvement in the research, and despite existing treatment for their condition—penicillin—they had been prevented from getting this treatment so that the research could continue. Lacks was an African American woman whose biospecimens were collected during a cervical cancer biopsy and later developed into the profitable HeLa cell line without her consent.^3^

See https://www.nihr.ac.uk/documents/the-nihr-carbon-reduction-guidelines/21685.

Holmes Rolston III discusses that obligations to protect non-human worlds are perhaps better understood at the species and ecosystem level.^30^ He also provides more detail on the various ways in which value is ascribed to non-humans.

In this report, sustainable development is defined as ‘meet(ing) the needs of the present without compromising the ability of future generations to meet their own needs’.

https://www.nihr.ac.uk/documents/the-nihr-carbon-reduction-guidelines/21685.

Also see Mancini *et al* 56 and Hickel *et al*.^57^

Open AI, “AI and Compute,” May 16, 2018, at https://openai.com/blog/ai-and-compute/.

Mainly for business reasons, but more recently to address considerations of the environment.^65^

https://www.ukbiobank.ac.uk/learn-more-about-uk-biobank/news/uk-biobank-creates-cloud-based-health-data-analysis-platform-to-unleash-the-imaginations-of-the-world-s-best-scientific-minds.
